# Electronic thermal conductivity in 2D topological insulator in a HgTe quantum well

**DOI:** 10.1038/s41598-018-36705-5

**Published:** 2019-01-29

**Authors:** G. M. Gusev, Z. D. Kvon, A. D. Levin, E. B. Olshanetsky, O. E. Raichev, N. N. Mikhailov, S. A. Dvoretsky

**Affiliations:** 1 Instituto de Física da Universidade de São Paulo, 135960-170 São Paulo, SP Brazil; 2 Institute of Semiconductor Physics, Novosibirsk, 630090 Russia; 3 Novosibirsk State University, Novosibirsk, 630090 Russia; 4 Institute of Semiconductor Physics, NAS of Ukraine, Prospekt Nauki 41, 03028 Kyiv, Ukraine

## Abstract

We have measured the differential resistance in a two-dimensional topological insulator (2DTI) in a HgTe quantum well, as a function of the applied dc current. The transport near the charge neutrality point is characterized by a pair of counter propagating gapless edge modes. In the presence of an electric field, the energy is transported by counter propagating channels in the opposite direction. We test a hot carrier effect model and demonstrate that the energy transfer complies with the Wiedemann Franz law near the charge neutrality point in the edge transport regime.

## Introduction

Two-dimensional topological insulators (2DTI) have attracted considerable attention in condensed matter physics for several reasons. Among them is the prediction of the existence of almost perfect edge channels in which electrons with opposite spin move in opposite directions without external magnetic field^1–5^. This hypothesis has been confirmed in micrometer-sized HgTe quantum wells from observation of the quantized local and nonlocal resistances^6–8^. Furthermore, the signature of the ballistic quantum transport is robust in InAs/GaSb quantum wells^9–12^ which is expected to be another variety of a two-dimensional topological insulator^13^. Another interesting prediction about topological insulators is the protection of the helical edge states against elastic backscattering by time reversal symmetry^1–5^. However, this remarkable property has not yet been experimentally confirmed^9–15^ and identifying the basic mechanism responsible for the backscattering at the edges of the topological insulators remains an important unresolved issue.

Application of novel experimental methods for the investigation of the transport properties of 2D TI is of particular interest.

Thermal conductivity measurements in metals and semiconductors have been often used as a powerful tool for probing transport mechanisms. Within the Landau Fermi liquid theory, electrons carry both charge and heat, and the relationship between thermal *κ* and electrical *σ* conductivities is known as the Wiedemann-Franz (WF) law, ${\mathcal L} =\kappa /\sigma T$. The Lorentz number takes the universal value ${ {\mathcal L} }_{0}=({\pi }^{2}/3)\,{({k}_{B}/e)}^{2}$, where *k*_*B*_, *e* are the Boltzmann’s constant and the electron charge, robust at low temperature and valid even in the presence of arbitrary disorder, when the Sommerfeld expansion can be applied. Deviation from the Wiedemann-Franz law at low temperature in the one dimensional (1D) case is associated with failure of the Fermi liquid model^16–20^. In particular, Luttinger liquid effects can be involved in the breakdown of the Wiedemann-Franz law citeli, houghton. However, at high temperatures, specific features of the Luttinger liquid effect begin to broaden and smear out, and a weakly correlated 1D electron system can be viewed, in a first approximation, as a Fermi liquid. The Wiedemann-Franz law has been verified in ballistic point contact^21^ and the experimental results demonstrate a satisfactory agreement both with the Kelvin-Onsager and Wiedmann-Franz relations. Helical edge states in topological insulators, however, have properties that are radically different from those expected in conventional 1D systems. For example, they have a linear dispersion, and, therefore, the inelastic electron-electron scattering within a single edge channel is much stronger than the transitions between the counter propagating edge states (which are rare because of the topological protection). This leads to counter propagating heat currents and different carrier thermalization and temperature profiles^22^.

**Figure 1:**
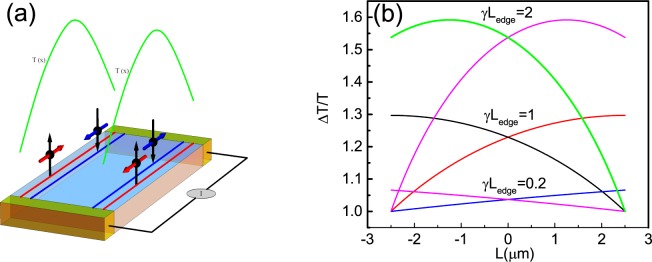
Schematic drawing of the slab shape sample with counter propagating spin polarized edge states and the electron temperature profile near the edge in the diffusive regime. The temperature profile of the helical states is calculated from Eq. 1 for quasiballistic transport and for different parameters of *γ* (see text for explanation).

In the present article, we report an experimental study of the electronic thermal conductivity in band-inverted HgTe-based quantum wells. At the charge neutrality point, electron transport is dominated by the edge state currents because the local and nonlocal resistances in our samples are comparable^14,15^. We measure non-linear voltage-current characteristics at different temperatures, and extract the electron temperature generated by Joule heating using the 2D TI temperature dependent resistance as its own thermometer. Assuming that the dissipation of Joule energy in the short samples occurs through the contacts rather than via phonon emission, the resulting electronic temperature distribution is universal and determined by the heating voltage and sample geometry^23^. Figure 1 shows the temperature profile due to Joule heating for one channel in accordance with the WF law for diffusive transport. One can see an inhomogeneous, parabolic profile of the electronic temperature along the boundary. An unusual temperature distribution appears in the two-dimensional topological insulators, where counter propagating electro-thermal flow is expected near the boundary. We show that overheating of the charge carriers leads to a nonmonotonic dependence of the differential resistance on the source-drain bias. We calculate the temperature profile in our samples and demonstrate that the results are correlated with the models proposed in^22^, describing different temperature profiles for the counterpropagating edge modes. The Wiedemann-Franz law is found to be valid near the charge neutrality point supporting the overheating of the edge state carriers model. Our findings pave the way for further exploration of quantized thermal transport in two-dimensional topological insulators.

## Results

**Figure 2:**
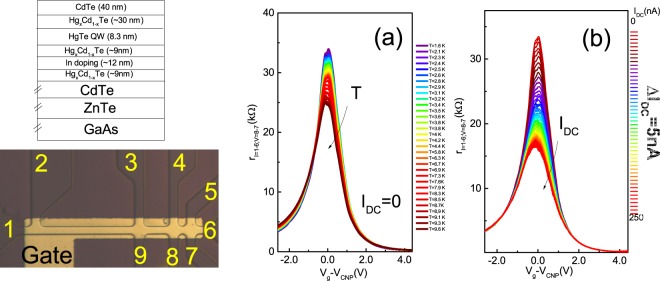
(**a**) The differential resistance as a function of gate voltage and bath temperature. (**b**) The differential resistance as a function of gate voltage and the DC current. Left bottom- schematic structure of the sample. Right bottom - top view of the sample.

The *Cd*_0.65_*Hg*_0.35_*Te*/*HgTe*/*Cd*_0.65_*Hg*_0.35_*Te* quantum wells with (013) surface orientations and width *d* of 8–8.3 nm were fabricated by molecular beam epitaxy (Fig. 2, left bottom panel). A detailed description of the sample structure has been given in paper^14^. The device is designed for multiterminal measurements and consists of three 3.2 *μm* wide consecutive segments of different length (3, 9, 35 *μm*), and 7 voltage probes (Fig. 2, right bottom panel). The ohmic contacts to the two-dimensional gas were formed by the in-burning of indium. To fabricate the gate, a dielectric layer containing 100 nm *SiO*_2_ and 200 nm *Si*_3_*Ni*_4_ was first grown on the structure using the plasmochemical method followed by a TiAu gate with the dimensions 62 × 8 *μm*^2^. The density variation with gate voltage is (1.09 ± 0.01) × 10^15^ *m*^−2^ *V*^−1^. Three different devices were studied. Table 1 lists the devises, and in the following section we discuss the parameters.

**Table 1:** Parameters of the electron system in HgTe samples at CNP, T = 4.2 K. Parameters are defined in the text.

Sample	*γL*_*edge*_	${\boldsymbol{ {\mathcal L} }}/{{\boldsymbol{ {\mathcal L} }}}_{0}$
1	2	1.1 ± 0.3
2	1.5	1 ± 0.3
3	1.1	1 ± 0.2

Below we show the results obtained in one representative sample 1 demonstrating quasi-ballistic behaviour. The differential resistance is studied when a current flows between contacts 1–6, and voltage is measured between probes 8 and 7, *r*_1–6,8–7_ = *dV*_8,7_/*dI*_1,6_. We employ lock-in technique at low-frequency (~4 *Hz*) using sufficiently small AC current to prevent self-heating (typically less than 1 nA). The differential resistance is measured as a function of the lattice temperature *T*_*L*_ and the DC current *I*_*DC*_ applied between the source and drain contacts.

The variation of differential resistance with gate voltage and lattice (bath) temperature is shown in Fig. 2a. Note that the minimum probe distance criteria are used to reduce the Joule heat dissipation via phonon emission. The resistance reveals a broad peak that is larger than the quantized value *h*/2*e*^2^ expected for the ballistic case. For comparison, in Fig. 2b, we show the variation of differential resistance with gate voltage and DC source-drain current. One observes a high similarity between the two plots: the zero bias resistance and the differential resistance strongly depending on temperature and *I*_*DC*_ respectively. Figure 2a,b contain individual curves of ${r}_{{I}_{DC}=0}(T)={r}_{0}(T)$ and *r*(*I*_*DC*_), allowing for a detailed comparison between them. Zero bias differential resistance decreases almost linearly with temperature *r*_0_(*T*) ~ *γ*(*V*_*g*_) × *T*, with the temperature coefficient *γ*(*V*_*g*_) strongly depending on gate voltage. Differential resistance depends in a similar way on the bias current. It decreases with increasing *I*_*DC*_, with a nonlinear coefficient depending on *V*_*g*_. The similarity between *r*_0_(*T*) and *r*(*I*_*DC*_) suggests that the nonmonotonic differential resistance results from a hot-carrier effect. It is instructive to linger on the possible mechanisms of the charge transport along the edge of the two-dimensional topological insulator. Observation of resistance higher than the quantized value in relatively long HgTe quantum well samples^6,8,14^ supports the notion that the topological protection in real samples is fragile due to disorder. There is an increasing number of models that are supposed to account for the deviation of the experimental resistance values from the expected quantized value^24–31^.

A promising model has been proposed quite recently^32^ claiming to account for the breakdown of the topological protection. It is argued that a realistic smooth edge potential results in a spontaneous breaking of the time-reversal symmetry responsible for the protection against backscattering. The edge reconstruction leads to a finite elastic scattering length, and conductance is described by the equation *G* = (*e*^2^/*h*)/(1 + *γL*_*edge*_), where *γ* is the inverse mean free path length for the edge to edge scattering, and *L*_*edge*_ is the effective length of the edge channels.

**Figure 3:**
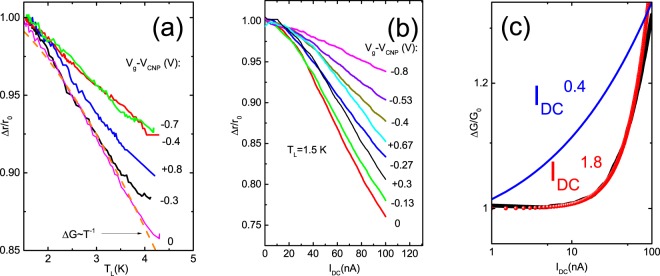
(**a**) Differential resistance as a function of lattice temperature for different gate voltages. Dashed line-linear T-dependence for the conductance. (**b**) Differential resistance as a function of DC current for different gate voltages. (**c**) Relative differential conductance as a function of *I*_*DC*_, *T*_*L*_ = 1.5 *K*.

Notice, however, that nonlinear transport is also one of the key features of a clean Luttinger liquid, due to the so called zero-bias anomalies resulting from the tunneling of electrons from the bulk to the wire. It is expected that differential resistance follows a power law *r* ~ *V*^*α*^ ~ *I*^*α*^, where *α* depends on the inter-electron interaction strength and the number of channels^17–19^. Figure 3c displays the relative differential conductivity as a function of *I*_*DC*_. One may see that *r*(*I*_*DC*_) shows a parabolic dependence rather than the bias dependence of $r\sim {I}_{DC}^{0.4}$ expected for a single channel. Note, however, that the observation of the zero-bias anomaly requires very low temperatures because it scales as a function of *eV*_*DC*_/*kT* and, at high temperature, this term becomes smaller than the hot carrier effect.

Before extracting the electron temperature, it is important to identify the mechanism that is responsible for the temperature dependence of zero-bias resistance. Firstly, one may suggest that it is a weak localization effect in one dimensional wires^33^. Note, however, that one-dimensional Wiedemann-Franz law theories are valid when the sample resistance is much larger than *h*/*e*^2^ (this is not the case for our sample when the shortest *L*_*edge*_ distance is used), and they were initially intended for conventional quantum wires (not for the helical edge states, where the conventional weak localization mechanisms might not work). To our knowledge, there is no theory on the Wiedemann-Franz law in helical edge states. Furthermore, we tried to check if the law *G* = *G*_0_ − Δ*G*, where Δ*G* ~ *T*^−1^ is the conductance of the short channel, can account for the 15% linear resistance decrease observed in the interval of temperatures from 1.5 K to 4.5 K (Fig. 3a). Comparing this law with the experimental results, we found an excellent agreement.

It is worth noting that we also measured differential resistance at higher temperatures. We found that the profile *r*_0_(*T*) above *T* > 15 *K* fits very well with the activation law *r*_0_ ~ *exp*(Δ/2*kT*), where Δ is the activation gap. The thermally activated behavior of resistance above 15 K corresponds to a gap of 10 meV between the conduction and valence bands in the HgTe well. The mobility gap can be smaller than the energy gap due to disorder.

Let us now address the transport properties of the helical edge states. The phase diagram for the conductivity of the helical liquid in the presence of the disorder and interaction has been considered theoretically in^19^. Three dominant scattering mechanisms have been taken into account. The first mechanism, the inelastic single particle scattering mechanism, denominated as a 1 P process, leads to a change in the chirality of a single incoming particle. The second important mechanism describes the inelastic backscattering of two electrons (2 P process). Finally, the authors argue that the backscattering may occur due to electron-electron interactions only^19^. The main results are summarized as follows:

1 $${\rm{\Delta }}G\sim \frac{{e}^{2}}{h}{L}_{edge}{T}^{-2K-2},K > 2/3$$

2 $${\rm{\Delta }}G\sim \frac{{e}^{2}}{h}{L}_{edge}{T}^{-8K+2},K < 2/3$$

where *K* is the Luttinger liquid parameter. It is important to note that, at *K* > 2/3, transport properties are dominated by 1 P scattering, and below *K* = 2/3, the 2 P process becomes important. In addition it is expected that, below *K* < 3/8, the localization of the helical edge states takes place. Assuming the Luttinger parameter *K* ≈ 3/8 (2 P process), which corresponds to the weak coupling regime, we obtain a good agreement with the experimental dependence ${\rm{\Delta }}G\sim \frac{{e}^{2}}{h}L{T}^{-1}$. Intriguingly, this parameter value also marks the transition from the localized to the delocalized regime in the transport of the disordered helical liquid. However, the study of the localization effects is out of scope of the present paper. It is worth noting that a previous study of strongly disordered topological insulators revealed a practically temperature independent resistance^14^ with, however, some tendency to localization behaviour.

It is also worth noting that the electron-phonon scattering predicted a *T*^3^ dependence of the scattering rate^34^, which disagrees with our observations.

As our sample resistance strongly depends on the electron temperature, this property can be used as a thermometer. Moreover, since the bulk conductivity is much smaller than the edge contribution (which is testified by a pronounced nonlocal effect^14,15^), the thermoconductivity must be dominated by the edge state transport and model^22^ is valid.

## Discussion

A quantitative check of the heat distribution in the sample provides further support for the hypothesis that the nonlinear effect results from Joule heating and electron thermal conductivity. A weak electron-phonon scattering is allowed in the presence of Rashba spin orbit coupling. However, because the sample is short, phonon emission does not occur at this distances (for details see Supplementary material). In addition, our samples are not ballistic as their peak resistance is larger than the quantized value (Fig. 2). Independently of a particular mechanism responsible for the backscattering (except for the electron-phonon coupling), in our samples, we obtain ${L}_{edge}\gamma \gg 1$ and we argue that the local temperature profile is controlled by the balance between Joule heating and electron thermal conductance. We argue that Joule heat is transferred by electrons and then dissipated via the electron diffusion into the bulk contacts outside the gate region. On the other hand, the inelastic electron-electron scattering within a single edge channel is free from these restrictions and, therefore, is much stronger. Moreover, such kind of scattering sweeps a large phase space, especially if the edge state velocity is energy-independent so that the momentum and the energy conservation laws are satisfied simultaneously for any two electrons participating in the collisions. Therefore, the temperature profile can be calculated, taking into account the boundary conditions *T*(0) = *T*(*L*) = *T*_0_ and Joule heat due to the current flow^22^:

3 $$\frac{{T}_{\pm }(x)}{{T}_{0}}=\sqrt{1+\frac{3}{{\pi }^{2}}[1+\frac{1\mp 2x/{L}_{edge}}{2/\gamma {L}_{edge}}]\frac{1\pm 2x/{L}_{edge}}{2/\gamma {L}_{edge}}{(\frac{{I}_{DC}}{{I}_{0}})}^{2}}$$

where *I*_0_ = *ek*_*B*_*T*_0_/*π*ℏ is the current associated with lattice temperature. Note that this equation has been derived within the approximation that all the heat is transferred by the electrons. In this case, the carrier thermal conductivity for each branch is given by *κ* = *γG*_*therm*_/2, where ${G}_{therm}=\frac{\pi {k}_{B}^{2}T}{6\hslash }$ is the quantized thermal conductance, and *k*_*B*_ is the Boltzmann constant^22^. Indeed, it was supposed that the ratio between the thermal and electrical conductivity for each branch obeys the Wiedemann Franz law $\frac{\kappa }{\sigma T}={ {\mathcal L} }_{0}$. The ratio $\frac{{I}_{DC}}{{I}_{0}}\sim \frac{G}{{G}_{therm}}$ can be used as an adjustable parameter in order to check the Lorentz ratio. The temperature profile strongly depends on parameter *γ*. Figure 1b shows the temperature difference along the sample for three different regimes: ballistic *L*_*edge*_*γ* = 0.2, quassiballistic *L*_*edge*_*γ* = 1, and intermediate between ballistic and diffusive *L*_*edge*_*γ* = 2 regimes and the same ratio $\frac{{I}_{DC}}{{I}_{0}}=1.5$. One can see that, due to the unidirectional character of thermal flow, for each mode, the temperature in each branch increases with distance. The heat is then dissipated in the contact region with the two-dimensional electron gas and, subsequently, transferred to the lattice. Therefore, the electron temperature abruptly decreases when the edge channel strikes the contact region outside the gate. In the ballistic regime, the temperature profile increases linearly with distance, while in the diffusive regime, the temperature profile has a parabolic shape^22^. In our samples *L*_*edge*_*γ* = 2, therefore, we expect a parabolic temperature profile.

From the temperature profile, we compute the average electron temperature as a function of the applied bias current:

4 $${T}_{e}({I}_{DC})=\frac{1}{{L}_{edge}}\,\int \,\frac{({T}_{+}(x)+{T}_{-}(x))}{2}dx$$

The low bias resistance versus lattice temperature dependence is thus used to determine the electron temperature *T*_*e*_ from the measured differential resistance *r*(*I*_*DC*_) versus applied bias current. However, we cannot discriminate between the temperatures *T*_+_ and *T*_−_ as we cannot discriminate between the contribution to the conductivity from each helical edge state.

The dependence of *T*_*e*_(*V*_*DC*_) is compared with our experimental data using Eqs (3) and (4). We perform curve fitting and extract the ratio of the experimental temperature to the average temperature found from Eqss (3) and (4) as a function of the gate voltage and the two temperatures. All approximation considered, the agreement is good. The dependence of *T*_*e*_(*V*_*DC*_) is compared with our experimental data using the Lorentz number as an adjustable parameter. The Table 1 shows the parameters *L*_*edge*_*γ* and ratio ${\mathcal L} /{ {\mathcal L} }_{0}$ for three different samples.

**Figure 4:**
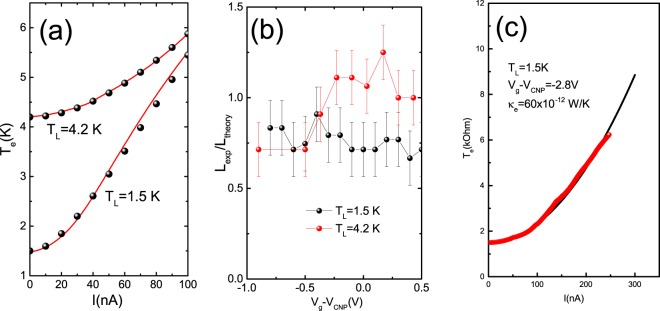
(**a**) Dependence of electron temperature, at the charge neutrality point, on the bias current for two lattice temperatures *T*_*L*_ = 4.2 and 1.5 K. Points-the averaged temperature found from eqs (1) and (2). (**b**) Dependence of the ratio of experimental electron temperature to the averaged temperature found from Equations (1) and (2) on the gate voltage for two lattice temperatures. (**c**) Dependence of electron temperature on the bias current in the bulk transport regime, ${\mathcal L} ={ {\mathcal L} }_{0}$.

Figure 4b shows the Lorentz ratio as a function of gate voltage for sample 1. The ratio ${\mathcal L} /{ {\mathcal L} }_{0}$ is close to one at both temperatures when the Fermi level stays in the insulating gap and transport is determined by the edge states. Good agreement with the Wiedemann-Franz law strongly supports the assumption that the thermal transport in a 2D topological insulator occurs via the edge states.

It is worth noting that in the bulk transport regime the temperature profile becomes universal and depends on the conductivity and the applied current:

5 $${T}_{e}^{2}={T}_{0}^{2}+\frac{3}{4{\pi }^{2}}{(\frac{e{I}_{DC}}{G})}^{2}(1-\frac{4{x}^{2}}{{L}^{2}})$$

where *L* is now the distance between probes. Indeed this universality is provided by the Wiedemann-Franz law^23^. The average temperature change is given by ${\rm{\Delta }}T=\frac{P{L}^{2}}{12{\kappa }_{e}}$, where *P* = *J*^2^/*σLW* (J is the current density, *σ* is the conductivity) is the Joule heating and *κ*_*e*_ is the bulk electron thermal conductivity.

We also studied the nonlinear differential resistance in the bulk transport regime *V*_*g*_ − *V*_*CNP*_ > |2.8 *V*|. As the temperature dependence of the resistance is very weak in this regime, the temperature distribution is described by Eq. 5. We recover the Wiedmann-Franz law, which gives support to our hypothesis that, for short distances, Joule heat is transferred only by electrons and justifies our analysis (Fig. 4c). Note that, for the bulk two-dimensional transport, the thermal conductivity is given by ${\kappa }_{e}=\frac{W}{12L}{G}_{th}$, where W is the width of the sample, and *G*_*th*_ is the thermal conductance.

In summary, we have measured the nonlinear differential resistance together with the low bias resistance in a 2D TI in HgTe quantum wells. We attribute the nonlinear effects to electron heating in the helical one dimensional edge states. We demonstrate that thermal electron conductivity is determined by edge state transport. By plotting the temperature profile as a function of the current bias, we extract the electron thermal conductivity near the CNP. The Wiedmann-Franz law is valid for both the edge states and the bulk transport regime.

## Methods

The samples studied here were grown by molecular beam epitaxy MBE^35^. A schematic section through the structure is shown in Fig. 2. Unlike the structures investigated previously^6,7^ in which a (100) surface was used for the MBE of the quantum wells, we have used a (013) surface. The quantum wells grown on it can have better quality than in the case of (100), and for that reason, we used a (013) surface in the present study^14,15^. The differential resistance was measured by use of an AC technique of applying a sinusoidal signal superimposed on a DC bias to the sample. Then a lock-in amplifier was used to obtain the AC voltage across and the AC current through the device. The nonlinear differential resistance was observed to decrease with increasing electrical bias current, (*dr*/*dI*_*DC*_ < 0), and was also observed to decrease with increasing lattice temperature (*dr*/*dT*_*L*_ < 0). Assuming that the change in the resistance with an applied electric field can be described in terms of electric field induced electron heating, temperature T in Eq. 2 in the main text can be replaced by the electron temperature *T*_*e*_. Therefore, *T*_*e*_ can be determined by comparing the relative resistances measured as functions of the lattice temperature *T*_*L*_ and the applied electric current *I*_*DC*_:

6 $${\rm{\Delta }}r({T}_{L})/r({T}_{{L}_{0}})={\rm{\Delta }}r({I}_{DC})/{r}_{0}$$

where ${T}_{{L}_{0}}=1.5\,K$ or 4.2 K. The electron temperature as a function of DC current was obtained from the curves shown in Fig. 3 of the main text.

## Electronic supplementary material

Supplemental Material
