# Supplementary material for: Electronic thermal conductivity in 2D topological insulator in a HgTe quantum well
Source: Sci Rep. 2019 Jan 29;9:831. doi: 10.1038/s41598-018-36705-5 (PMC6351662; doi:10.1038/s41598-018-36705-5)
Supplement: Supplementary file 1 — Supplemental Material [file 41598_2018_36705_MOESM1_ESM.pdf]

Supplemental Material : Electronic thermal conductivity in a 2D topological insulator in a HgTe quantum well

G. M. Gusev[1,*], Z. D. Kvon[2,3], A. D. Levin[1], E. B. Olshanetsky[2], O. E. Raichev[4], N. N. Mikhailov[2], and S. A. Dvoretsky[2]

[1]Instituto de Física da Universidade de São Paulo, 135960-170, São Paulo, SP, Brazil
[2]Institute of Semiconductor Physics, Novosibirsk 630090, Russia
[3]Novosibirsk State University, Novosibirsk 630090, Russia
[4]Institute of Semiconductor Physics, NAS of Ukraine, Prospekt Nauki 41, 03028 Kyiv, Ukraine
[*]gusev@if.usp.br

ABSTRACT

In this supplementary, we provide comparison with a model, taking into account the power loss via acoustic phonons.

1 Comparison with phonon emission model

In the main text, we assumed that the energy is dissipated in the bulk outside the gate region in the bulk contacts, where the electrochemical potential is mixed. Because the sample is short, the phonon emission does not occur at this distance. However, at high sample temperatures and for long samples, it is expected that the dominant cooling mechanism switches to electron-phonon coupling. In this regime, Joule heating follows to:

$$P = \Sigma_{ep}(T_e^\gamma - T_L^\gamma) \sim T_e^\gamma \tag{1}$$

where Σ_{ep} is the electron-phonon coupling parameter. Calculations of the energy loss rate P in the Bloch-Gruneisen regime in one dimensional wires has been performed in paper [1]. Electron scattering by three dimensional acoustic phonons through the piezoelectric field (PA) and deformation potential (DA) have been considered.

In this supplementary material, we perform calculations of the power loss due to phonons in our system, with transport dominated by one dimensional edge states along the border of HgTe based quantum wells. Results of such calculations are shown in Figure 1. The dashed line is calculated for the simple approximation $T << s\kappa$ (s -sound velocity, κ - inverse decay length of the edge-state wave function).

Note that the transition probability in the one-dimensional case is much smaller than in a two-dimensional system, and the emission rate or power loss via phonons approaches only pW values, while the maximum total Joule heat is near a few nW. Indeed such value is not sufficient for cooling, and the heat balance is described by diffusive cooling with a nonuniform temperature profile and energy dissipation in the leads (which may occur via phonons). Theory predicts the exponent of T, $\gamma \approx 4$, while in our experiment we found $\gamma \approx 2.8$. Note that a simple calculation in approximation $T << s\kappa$ predicts higher power loss, however, the exponent of T $\gamma \approx 5$, which disagrees with our observations.

Based on this argument we conclude that Joule energy in the short samples occurs through the contacts rather than phonon emission.

References

1. S. S. Kubakaddi, Phys. Rev. B 75, 075309 (2007).

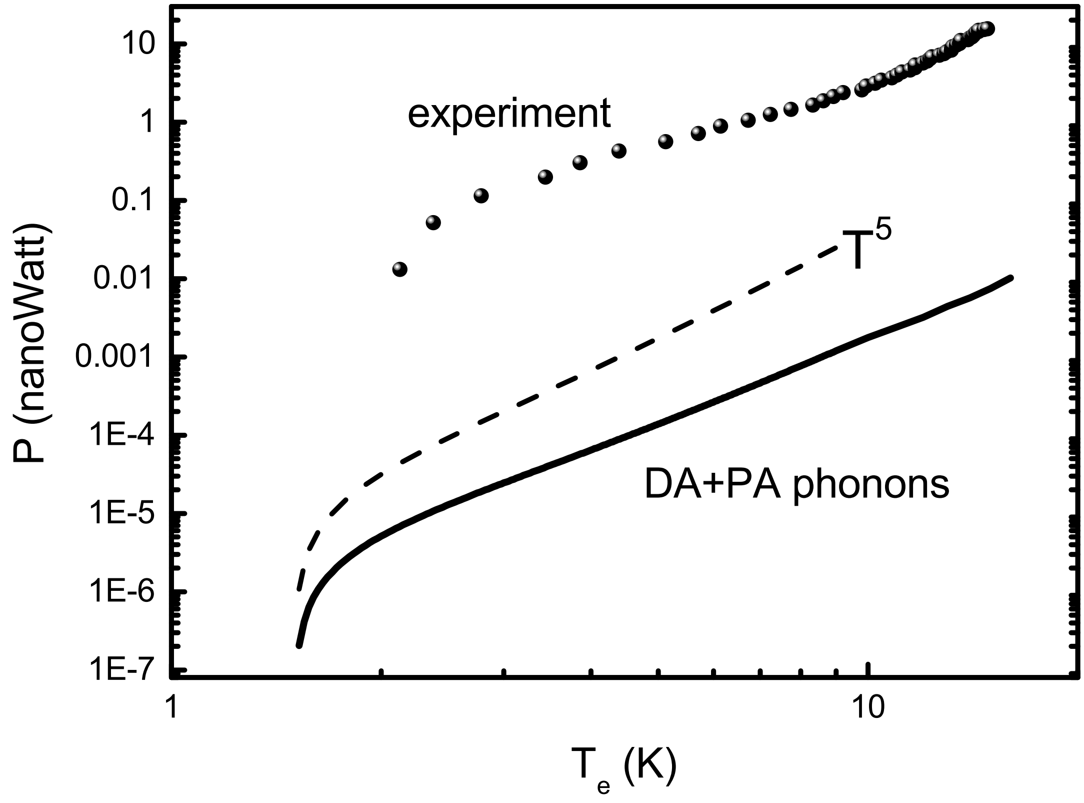

Figure 1. (Color online) The electron power loss P as a function of measured (dots) and calculated (solid line) electronic temperature T in edge states of a two-dimensional HgTe topological insulator for different electron-phonon scattering mechanisms. Dashed line represents the simple approximation $T << s\kappa$ (s -sound velocity, κ - inverse decay length of the edge-state wave function).
